# Prediction of the visit and occupy of the sika deer (*Cervus nippon*) during the summer season using a virtual ecological approach

**DOI:** 10.1038/s41598-023-31269-5

**Published:** 2023-03-10

**Authors:** Takeshi Osawa, Narumasa Tsutsumida, Hayato Iijima, Kimiko Okabe

**Affiliations:** 1 Graduate School of Urban Environmental Sciences, Tokyo Metropolitan University, Minami-Osawa 1-1, Hachioji, Tokyo 192-0397 Japan; 2 Department of Information and Computer Sciences, Graduate School of Science and Engineering, Saitama University, Saitama, Japan; 3 Forestry and Forest Products Research Institute, Matsunosato 1, Tsukuba, Ibaraki 305-8687 Japan

**Keywords:** Animal migration, Animal migration, Animal behaviour

## Abstract

Prediction of the spaces used by animals is an important component of wildlife management, but requires detailed information such as animal visit and occupy in a short span of the target species. Computational simulation is often employed as an effective and economical approach. In this study, the visit and occupy of sika deer (*Cervus nippon*) during the plant growing season were predicted using a virtual ecological approach. A virtual ecological model was established to predict the visit and occupy of sika deer based on the indices of their food resources. The simulation results were validated against data collected from a camera trapping system. The study was conducted from May to November in 2018 in the northern Kanto region of Japan. The predictive performance of the model using the kernel normalized difference vegetation index (kNDVI) was relatively high in the earlier season, whereas that of the model using landscape structure was relatively low. The predictive performance of the model using combination of the kNDVI and landscape structure was relatively high in the later season. Unfortunately, visit and occupy of sika deer could not predict in November. The use of both models, depending on the month, achieved the best performance to predict the movements of sika deer.

## Introduction

Prediction of the spaces used by animals is an important component of wildlife management, conservation, and population health^1,2^. However, wildlife managers often face complex challenges to predict the spaces used by large animals, especially migratory mammals because these species often travel over long distances through various types of habitats^3,4^. The space used by such animals may not be stable, not determined by the single types of habitats. Therefore, large-scale management of a target species of migratory mammals could also include maintaining the quality of different types of habitats^3,4^.

Additionally, animal migrations can be classified into at least two types: (1) generally occur within a particular home range, and (2) occasionally occur beyond the home range^5^. Some animals engage in periodical round trip movements between seasonal home ranges^6–8^. For example, seasonal migration from high elevations to mid- or low elevations are common for ungulates, such as the sika deer (*Cervus nippon*)^6,9,10^, roe deer (*Capreolus capreolus*)^11^, takin (*Budorcas taxicolor*)^8^, and moose (*Alces alces*)^12^. Some species of ungulates are known to exhibit large-scale movements within a particular season (type 1) and between seasons (type 2).

Among ungulates, the overabundance of deer populations in many regions negatively impact ecosystems and human society due to over browsing and trampling of vegetation, deer-vehicle collisions, and transmission of wildlife diseases, including zoonoses^13–15^. Accordingly, wildlife managers often employ lethal control measures (e.g., culling) to reduce the negative social and ecological impacts of deer^16–18^. However, lethal control measures affect the behavior and habitat utilization of deer populations, resulting in learned avoidance^18–21^. Therefore, from the perspective of management intervention, wildlife managers should predict the areas that deer tend to visit and occupy in order to concentrate management efforts in a particular area^18^.

Prediction of the areas that animal visit and occupy requires investigations of the association between the species and habitat by transect surveys and biologging methods^22^. However, the collection of such detailed data of animal is limited by cost^23^. To address economic restraints, computational simulation is an effective tool that allows for explicit representation of basic information of large-scale animal visit and occupy patterns without detailed observation efforts^24^. Hence, the simulation approach has been extensively employed to predict the visit and occupy of migrating animals^24^.

Camera traps are becoming increasingly popular survey instruments for non-invasive studies of various animal behaviors^25,26^. This method offers a reliable, minimally invasive, visual means to survey wildlife, while substantially reducing survey efforts^25^. However, the use of camera traps at the landscape scale requires a well-designed setting, a sufficient number of cameras, the availability of researchers to place and check the cameras, and data processing^25,27^. Here, we propose a combination idea using a computational simulation approach with minimal, not well-designed camera traps. We used a virtual ecology approach that simulates species dynamics using simple models together with virtual observation of the simulation result^28,29^. Using this approach, a hypothetical simulated dispersal of the target species can be evaluated using actual observation records, even with limited^30–33^. The framework of the proposed virtual ecological approach allows us to use limited camera trap data effectively to reflect the actual dynamics patterns of the target species.

In the present study, a virtual ecology approach was used to predict the visit and occupy of sika deer during the plant growing season. Although sika deer migrate between the summer and winter seasons^6,10,34^, this study focused on the summer season based on the driving factors of visit and occupy, especially the availability of food resources, which is the most important factor triggering migration of large herbivores^5,7,9,11^. The sika deer tend to visit and occupy areas within a home range with high accessibility to food resources. Data to reflect the amount of food resources of deer are acquired from remote sensing measurements^35,36^ and landscape indices^37,38^. The designated hunting season within the study area extends from November to February (https://www.pref.tochigi.lg.jp/d04/syuryouseido.html, accessed on Feb. 10, 2023), denoting the winter season, however, supplemental hunting sanctioned by the governmental authorities was carried out during the summer season (https://www.pref.tochigi.lg.jp/d04/eco/shizenkankyou/shizen/documents/shika6.pdf, accessed on Feb. 10, 2023). Furthermore, sika deer can transmit zoonosis during the plant growing season through ticks as vectors^39^. Thus, the prediction of the visit and occupy of sika deer during the plant growing season (i.e., May to November in the north Kanto region of Japan) is significant. We predicted the visit and occupy of sika deer in the summer season using two candidate proxies to reflect the availability of food resources. Subsequently, the simulation outputs of the camera trapping results were used to evaluate the accuracy of the simulation results. Finally, the usefulness of the proposed virtual ecological approach for deer management is discussed.

## Results

The camera trapping system included 14 cameras, which included seven for capturing images of the sika deer every month (Table 1). However, one of the cameras failed to capture images for a period of 7 months (Table 1).

**Table 1:** Occurrence of captured sika deer in each camera trap over a period of 8 months.

Camera ID	May	Jun	July	Aug	Sep	Oct	Nov	Total
1	1	1	1	1	1	1	1	7
2	1	1	1	1	1	1	1	7
3	0	0	1	1	1	1	0	4
4	1	1	1	1	1	1	1	7
5	1	1	1	1	1	1	1	7
6	1	1	1	1	1	1	1	7
7	1	1	1	1	1	1	0	6
8	1	1	1	1	1	1	1	7
9	1	1	1	1	1	1	1	7
10	0	1	1	0	0	0	0	2
11	0	1	1	0	0	0	0	2
12	0	1	0	0	0	0	0	1
13	0	1	0	0	0	0	0	1
14	0	0	0	0	0	0	0	0

The proportions of “correct” runs for every 100 runs each month are shown in Table 2. All simulation results excluded both June and November were greater than that of the equivalence model (Table 2). More than 80% of the kNDVI values for May, July, and August were “correct”, while 41 values for October were “correct” and almost all values for June, September, and November were not decided as “correct” (Table 2). In contrast, with the exception of June and November, the landscape structure model was considered “correct” for most of the study period (Table 2). The results of the AND and OR simulations were basically the same as the results of the kNDVI and landscape structure models.

**Table 2:** Simulation results of the number of “correct” for each model. In total, 100 runs were conducted for each simulation. The best performance for each month is highlighted by bold italic text.

Month	kNDVI	Landscape	AND	OR	Equivalence model
May	88	***100***	57	***100***	53
June	1	0	4	0	***9***
July	82	***100***	47	***100***	47
August	90	***100***	61	***100***	79
September	0	***100***	0	***100***	79
October	41	***100***	12	***100***	79
November	2	0	3	0	***47***

**Figure 1:**
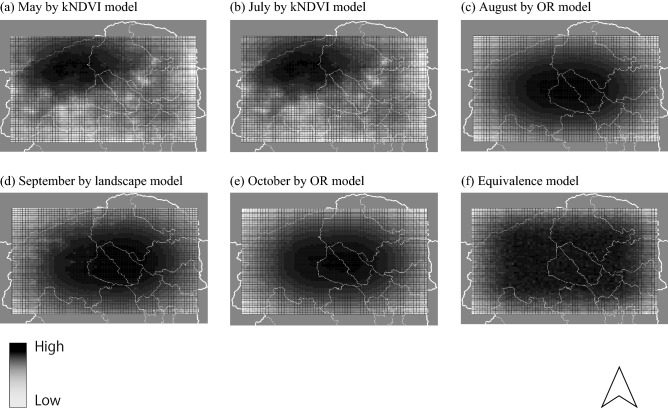
Simulation results of the best predictive performance among all simulations vs. the equivalence model. Deep black indicates a high frequency of visits by the virtual population. Local governmental borders are indicated by white lines. The results of June and November are not shown because the simulation model could not predict the movements of sika deer during these months.

The results of the predictive ability are shown in Table 3. The predictive ability of the kNDVI model was optimal in May and July for all four simulations (Table 3). The best predictive ability of the landscape model for all four simulations occurred in September (Table 3). For the OR model, the best predictive ability for all four simulations was in August and October (Table 3). The best predictive ability model in both June and November was not determined because the number of “correct” results was lower than that of the equivalence model (Tables 2, 3). From May to October, with the exception of June, the equivalence model and map of the number of theoretical visits by sika deer were based on the best results in Fig. 1.

**Table 3:** Predictive ability of the simulation results. The number that the model considered as “correct” and showed the lowest AIC among 4 models was shown. In total, 100 runs were conducted for each simulation, but some of the runs were not considered as “correct”, thus, the total number was not always 100. The best performance for each month is highlighted by bold italic text.

Month	kNDVI	Landscape	AND	OR
May	***69***	0	21	10
June	1	0	***4***	0
July	***55***	0	13	32
August	37	0	10	***53***
September	0	***77***	0	23
October	1	2	3	***94***
November	2	0	***3***	0

## Discussion

In this study, the movement simulation data of sika deer during the plant growing season were validated against the data obtained with the camera trapping system. The predictive performance of the simulation results using the kNDVI to track the movements of sika deer in the earlier months was relatively high, whereas that of the landscape structure was relatively low. The predictive performances of the OR model in later months were relatively high. Therefore, use of both the kNDVI and landscape structure can accurately predict the monthly movements of sika deer at some degrees. Hence, this approach could help to establish new management strategies.

Simulations with both the kNDVI and landscape structure models exhibited high predictive ability as compared to the equivalence model with the exclusion of data from both June and November. All simulation results, including the AND and OR models, failed to predict the movements of sika deer in June and November. For the month of June, a possible explanation for the failure of the models is the breeding season of sika deer. Female sika deer give birth from mid-May with a peak in June^40^, and female adults have high pregnancy rates^40^. During the fawning season, the movements of females are decreased in lieu of safe sites with available food resources. Only 4 of the 14 cameras captured images of a sika deer in June and/or July. Thus, these 4 cameras were likely placed in safe sites for sika deer. If the simulation model improves to differentiate between males and females, the overall predictive ability of the simulation could significantly improve. This point constitutes a crucial challenge for the next step in this study. In November, the designated hunting period starts in the study area (https://www.pref.tochigi.lg.jp/d04/syuryouseido.html, accessed on Feb. 10, 2023), which could cause changing behavior and habitat utilization of deer populations^18–21^. This factor may have contributed to the comparatively heightened simulation performance of the equivalence model in November.

The kNDVI model, which had the best predictive performance for all simulations, successfully predicted the movements of sika deer in both May and July, likely because of the inclusion of vegetation conditions as potential food resources^41^. However, the kNDVI only reflects the condition of the surface layer of vegetation. Therefore, if the target area is covered by a tree canopy, the kNDVI cannot reflect the conditions of grasses and other plants or seedlings on the forest floor, which are often preferred by sika deer^14^. Actually, the predictive performance of the kNDVI model had decreased after August, possibly due to the loss of the tree canopy in the study area, which is a limitation to satellite remote sensing instruments to predict the condition of ground vegetation.

The landscape structure model accurately predicted the movements of sika deer virtually throughout the study period, with the exception of June and November. These results were in agreement with those of previous studies that the forest edge has an abundance of food for sika deer^37,38,42,43^. Thus, the landscape structures of forests and grasslands combined could reflect food resource availability for sika deer. However, the predictive performance of the landscape structure model was relatively low, with the exception of September. In this study, the predictive performance of the landscape structure model was limited by seasonal change because of the inability to reflect changes to vegetation. However, the landscape structure model showed the best predictive performance in September. As a possible explanation is their stability of food resources. The seasonal rutting of sika deer begins in September^40^, thus many individuals might visit a particular area in search of a mate. The areas with modest but stable food resources might have frequent visiting by sika deer. To clarify the underlying causes of this seasonal pattern, biologging methods could be employed for detailed monitoring of sika deer.

The best predictive performance in both August and October was obtained with the OR model. In theory, the OR model could complement the performance the kNDVI and landscape structure models. The predictive performance of the kNDVI model was improved in the early season due to the capability to weigh the conditions of grasses and other plants of seedlings as available food resources for sika deer. However, the landscape structure model could also reflect the availability of food resources that are not influenced by seasonal change. Due to the combination of these characteristics, the OR model can accurately predict areas with grasslands neighboring forests. After the summer season, such areas might have abundant food resources for sika deer.

## Conclusion

The proposed virtual ecological approach based on food resource availability was useful to predict the movements of sika deer during the plant growing season, at least to some extent. Importantly, the predictive performances of simulation models are improved by the inclusion of seasonality of vegetation, such as the kNDVI. Thus, the virtual ecological approach should prove useful to wildlife managers to predict target zones for management of sika deer, although the timing of implementation should be considered for the type of simulation model. Nonetheless, the virtual ecological approach can improve management of sika deer.

## Methods

### Study area and camera trapping system

**Figure 2:**
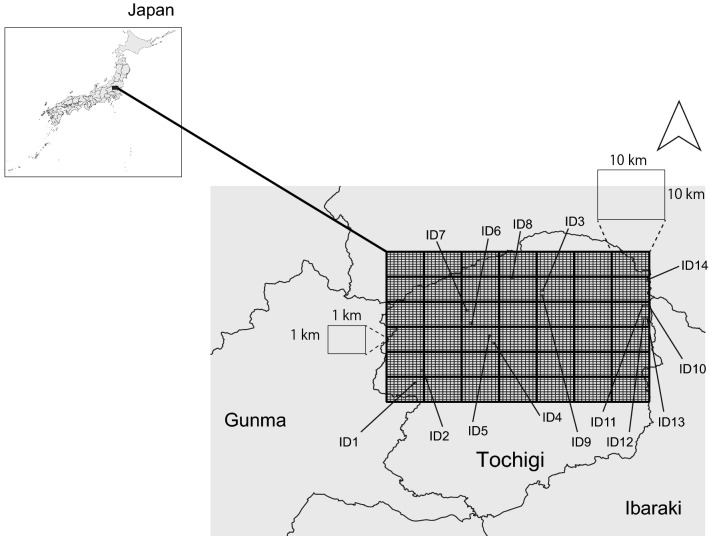
Study area, analytical units, and locations of the camera traps.

The study area included the northern region of Tochigi Prefecture, Japan (Fig. 2). In Tochigi Prefecture, 54.4% of the land was covered by forest, 19.1% was covered by agricultural land in 2019 (Tochigi Prefecture 2021, https://www.pref.tochigi.lg.jp/a03/documents/keikakusho2267.pdf, accessed on Feb. 10, 2023). The northern region of Tochigi Prefecture has a relatively large area of forest. This area was the home range of the highest density of sika deer in Tochigi Prefecture in 2021. The camera trapping system consisted of 14 cameras (model no. 6210; Ltl-Acorn, Des Moines, IA, USA) that were placed in late April 2018 at 12 sites within the forest interior with two camera sets, namely ID 10–11, and ID 12–13 in neighboring areas (Fig. 2). The 12 sites spanned 84 km from west to east and 39 km from north to south (Fig. 2). The elevation of the sites ranged from 349 to 1033 m. The cameras were set horizontally at 50 cm above the ground and were operated until late November 2018. The cameras were checked every 1 or 2 months and the batteries and memory cards were replaced when necessary. Movements of the sika deer were reordered monthly from May to November. The month of April was excluded because the cameras were placed in late April. The virtual ecological model required the presence/absence of records for validation (described below), thus the number of deer captured in the photos was not considered. Finally, the visit and occupy of sika deer were recorded at 14 sites each month.

A grid size of approximately 1 km (termed “1-km mesh” hereafter) was used a as the study unit (Fig. 2). The 1-km mesh grid system is a standard Japanese unit used for several types of statistics (https://www.stat.go.jp/english/data/mesh/02.html, accessed on Feb. 10, 2023). To determine the appropriate number of 1-km mesh grids for the simulation study, a 10-km mesh grid, which is the high-order standard Japanese unit (i.e., one 10-km mesh includes 100 1-km meshes), was divided into the minimum number of areas to cover all 14 camera sites as the simulation target area to avoid arbitrary (Fig. 2). Finally, 4200 1-km mesh areas were included for the simulation (Fig. 2).

### Virtual ecological model

**Figure 3:**
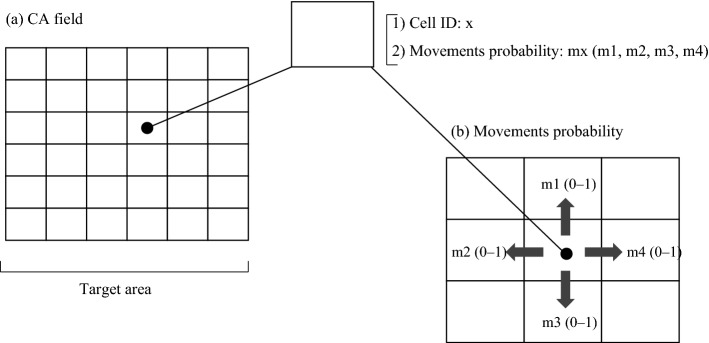
Basic structure of the cellular automaton model. (**a**) Two values are associated with each cell: the cell ID “x,” a unique ID for each cell, and the movement probability “mx” indicating four directional vectors into adjacent cells. (**b**) Values m1, m2, m3, and m4 indicate the probability of movement along a path of the top, left, bottom, and right cells, respectively. If all movement probability values are 0, the virtual population in this cell cannot move to any other cell. If all movement probability values are 1, the virtual population in the cell can move to all adjacent cells.

A simple cellular automaton (CA) model can predict the visit and occupy of a target species based on candidate habitats in consideration of the proximity to food resources^32^. The grid was set to the same size as the unit of the predicted ranges. The model yields a theoretical number of visits (described below) to each cell, which serves as an area preference of the target species. Each cell has two parameters: cell identification (ID) and movement path vector (Fig. 3a). The cell ID indicates the spatial location of the cell within the study area. The movement path involves four variables representing the four directional vectors into adjacent cells (i.e., top, left, bottom, and right) (Fig. 3b). Each variable is a probability value (i.e., 0 to 1) independent of the other three variables that indicates the probability of movement success to the adjacent cells. In this study, the probability value was based on the proximity to availability food resources.

A group of sika deer was used as the unit for analysis. The model simulates the capability of movement within the target area. Thus, if a virtual population visited a neighboring cell, the number of visits to the cell is increased without disappearance of the starting cell. The virtual population moves in accordance with the movement probability values.

### Movement probability between cells

The term “movement probability” is defined as the probability of movement success into an adjacent cell to the top, left, bottom, or right (Fig. 3b) with four probability values:

1 $$ {\text{Movement probability x}} = {\text{mx}}\;({\text{m}}1,\;{\text{m}}2,\;{\text{m}}3,\;{\text{m}}4), $$

where m1, m2, m3, and m4 indicate the probability of movement success into the top, left, bottom, and right cells, respectively (Fig. 3b). Since these values are independent of one another, the maximum and minimum sums of m1, m2, m3, and m4 are theoretically 4 and 0, respectively. If all probability of movement success values are 0, the sika deer population in this cell cannot move to any other cell. Moreover, if all probability of movement success values are 1, the population in the cell can move to all adjacent cells.

The amount of food resources of deer was acquired from remote sensing measurements^35,36^. Thus, two variables were used to represent food resource availability: the kernel normalized difference vegetation index (kNDVI)^41^ and landscape structure (Supplementary Fig. 1).

The kNDVI uses remote sensing measurements to assess the components of green vegetation^41^. As compared to the ordinal NDVI, which is the most widely used index of the condition of vegetation on terrestrial surfaces, the kNDVI has greater resistance to saturation, bias, and complex phenological cycles, and exhibits enhanced robustness to noise and stability across spatial and temporal scales^41^. The kNDVI appropriately represents the condition of vegetation to reflect the food resource availability for sika deer. The kNDVI was analyzed from the atmospherically corrected surface reflectance observed with the Landsat 8 Operational Land Imager and Thermal Infrared Sensor instruments at approximately 16-day intervals with a spatial resolution of 30 m (data collected in 2018). The mean kNDVI was calculated monthly for each 1-km mesh within the study area. The probability values (m1, m2, m3, and m4) were defined as the proximity to available food resources in a destination cell divided by the maximum value of the target area as relative values throughout the study area. These values reflect the spatial positions of the available food resources in the study area. If the food resources are continuously available, then the sika deer population tend to visit and occupy linearly.

The landscape structure is defined as a mixture of forests and grasslands because previous studies suggest that the forest edge has high availability of food resources for sika deer^37,38,42,43^. The dataset was generated from a current vegetation map that classified the dominant plant species provided by the Biodiversity Center of Japan (Ministry of the Environment, https://www.biodic.go.jp/index_e.html, accessed on Feb. 10, 2023). The types of vegetation of the forests and grasslands were retrieved from the literature, then the original vegetation classes were re-classified^44^ and overlayed on the 1-km mesh map. In this study, agricultural land types were classified as grassland. For a mesh with both forests and grasslands, the probability of movement was assigned a value of 1, while a mesh with either a forest or grassland was assigned a value of 0.5, because to treat these 2 components fairly. Every mesh of the study area included either a forest or grassland.

### Movement simulation

First, simulations were conducted using two independent variables: kNDVI and landscape structure. Each simulation was initiated from one cell with the month, which is referred to as a “trial.” One step is defined as one day, thus the trial conducted in May consisted of 31 steps. A previous study reported that sika deer can travel about 50 km every 2 weeks^34^. Thus, one step (movement of 1 km) in one day was considered a reasonable distance. Each trial was repeated for all cells i.e., all cells was used as the starting cell of “trial”. The sum of all trials is termed a “run.” Thus, each “run” consisted of *n* trials, where *n* is the number of cells in the CA field. In this study, there were 4200 cells. At each step, each attempt to visit a neighboring cell (top, left, bottom, and right) was based on movement probabilities. For each successful movement, the presence/absence value assigned to the cell was increased from 0 to 1, i.e., change from absence to presence. The next step was then initiated from any newly visited cell and the previously visited cells. Cells with high values indicated the possibility of visitation by a virtual population from several other cells. The assigned value was used as a metric of the preference of the visited cell. In this study, 100 runs were conducted each month from May to November.

Second, simulations were conducted using a combination of movement-related variables with two types of combination models: kNDVI AND landscape structure and kNDVI OR landscape structure. With both the logical AND and OR models, each step has two processes: probability approach with the kNDVI and landscape structure. With the AND model, if the virtual population passes the probability of the kNDVI to move to a neighboring cell, then the probability of movement to a neighboring cell is based on the landscape structure. In the logical AND model, we used kNDVI first because that could reflect a seasonal change in the availability of food resources. With the OR model, if the virtual population passes the probability of the kNDVI, or passes that of the landscape structure, the virtual population can move to any neighboring cell.

Additionally, equivalence model simulation was conducted with all probability values (m1, m2, m3, and m4) set to 0.5.

### Validation of the simulation results using the camera trap data

The results of the CA model simulation were validated by the presence/absence of the monthly records of sika deer collected with the cameras. The occurrence of a visit to a camera was determined using a generalized linear model with a binomial distribution (log link) and model selection based on Akaike’s information criterion (AIC). The explanatory variable was the theoretical number of simulated visits to a 1-km cell with a camera trap. If the AIC value of the model was > 2 points lower than that of the null model^45^ (i.e., with no explanatory variable), the run was considered “correct”. The data from the kNDVI, landscape structure, AND/OR, and null/equivalence models were used. The number of “correct” runs of every 100 runs with each model was calculated. Therefore, all values could theoretically be 100.

Then, the predictive ability of the model was evaluated using the results considered as “correct” with the AIC. The AIC values of all runs were compared, where one simulation set used four variables. If the four models (i.e., kNDVI, landscape, AND, and OR models) were all “correct” in one run, the AIC values were compared and the lowest AIC value of the model was recorded. Notably, differences among the AIC values were not considered because the effectiveness of the model was already evaluated in the first validation procedure. Calculations for all months were conducted. Therefore, the maximum value among the four models was 100, assuming that the run was “correct” with the lowest AIC.

Finally, a map was generated of the theoretical number of visits by sika deer in each month based on the best performance among the four simulations. The map included the average number of theoretical visits over 100 runs. The results considered incorrect were not excluded because in real-world applications, simulated results are not evaluated.

All statistical analyses were performed using R software (ver. 4.0.2; https://www.r-project.org/, accessed on Feb. 10, 2023).

## Supplementary Information

Supplementary Figure 1.

Supplementary Legends.

## Data Availability

Original data are available in the text and from for corresponding author upon reasonable request.
