# Supplementary material for: Prediction of the visit and occupy of the sika deer (Cervus nippon) during the summer season using a virtual ecological approach
Source: Sci Rep. 2023 Mar 10;13:4007. doi: 10.1038/s41598-023-31269-5 (PMC10006405; doi:10.1038/s41598-023-31269-5)

Osawa et al. Appendix Fig. 1

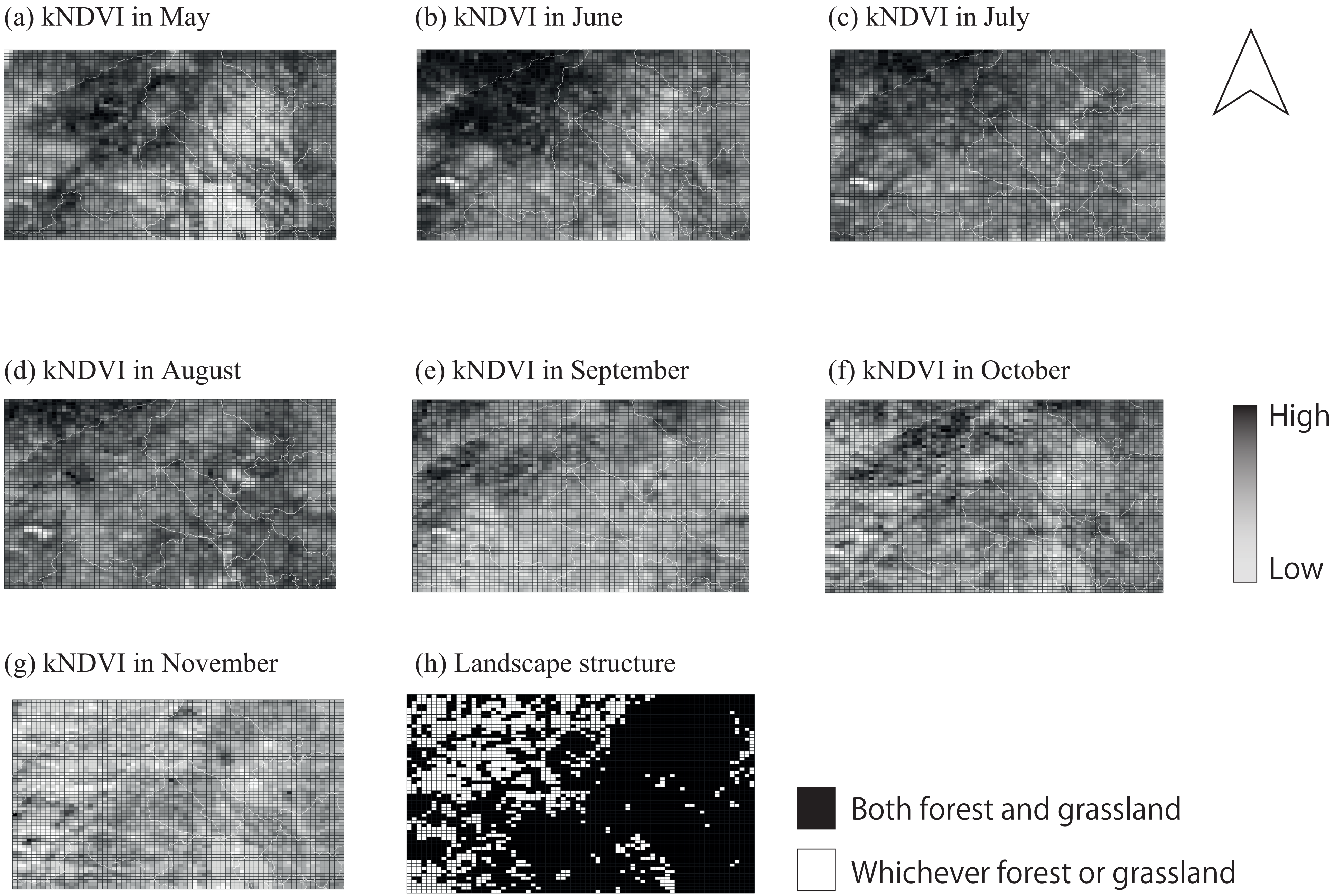

Supplement: Supplementary file 1 — Supplementary Figure 1. [file 41598_2023_31269_MOESM1_ESM.pdf]
