# Supplementary material for: Prediction of the visit and occupy of the sika deer (Cervus nippon) during the summer season using a virtual ecological approach
Source: Sci Rep. 2023 Mar 10;13:4007. doi: 10.1038/s41598-023-31269-5 (PMC10006405; doi:10.1038/s41598-023-31269-5)
Supplement: Supplementary file 2 — Supplementary Legends. [file 41598_2023_31269_MOESM2_ESM.docx]

Appendix Fig. 1. A map of two variables representing the availability of food resources for deer: kNDVI and landscape structure. Local governmental borders are indicated by white lines.
